# Hypertension as an effect modifier for preterm and small for gestational age births in migrant women in Belgium: A population-based study

**DOI:** 10.1371/journal.pone.0323652

**Published:** 2025-05-14

**Authors:** Clotilde Lamy, Amira Doghri, Elena Costa, Michel Boulvain, Alice Hocquette, Sophie Alexander, Judith Racapé

**Affiliations:** 1 Hôpital Universitaire de Bruxelles (HUB) - Hôpital Érasme, Université Libre de Bruxelles (ULB), Clinique de gynécologie obstétrique, Brussels, Belgium; 2 Research Center in Epidemiology, Biostatistics and Clinical Research. School of Public Health, Université Libre de Bruxelles (ULB), Brussels, Belgium; 3 Belgian Health Care Knowledge Centre, Brussels, Belgium; 4 Department of Obstetrics and Prenatal Medicine, University Hospital UZ Brussel, Vrije Universiteit Brussel (VUB), Brussels, Belgium; 5 Université Paris Cité, CRESS, INSERM, INRA, Paris, France; 6 Faculty of Medecine, Université Libre de Bruxelles (ULB), Brussels, Belgium; University of Campinas, BRAZIL

## Abstract

**Background:**

The association between migration and pregnancy outcomes gives contradictory results. Women’s socio-economic status explains some differences, but its influence may vary according to women’s underlying health conditions. Our aim was to understand how comorbidities modify the relationship between migration and preterm birth or small for gestational age in Belgium.

**Methods:**

Data are related to all singleton births to women living in Belgium between 2010 and 2019 (n = 1 200 417). Maternal nationalities were grouped as Belgium, European Union, Eastern Europe, North Africa, Sub-Saharan Africa and the Middle East. A logistic regression was used to estimate the association between maternal nationalities and perinatal outcomes, taking into account the socio-economic status and maternal comorbidities: hypertension, obesity, and diabetes. The interaction effect between maternal nationalities and comorbidities was tested.

**Results:**

Migrant women were more socio-economically disadvantaged than Belgian women. All migrant women without hypertension had a significantly lower Odd Ratio of preterm birth and small for gestational age than Belgian (p < 0.001). In contrast, women with hypertension had a higher OR than Belgian women, even after adjustment for socio-economic status and other comorbidities. This difference was more striking among Sub-Saharan African and Middle Eastern women: respectively, aORs 1.45 (95%CI 1.30–1.62) and 1.24 (95%CI 1.01–1.54) for preterm birth, and aORs 1.17 (95%CI 1.03–1.17) and 1.28 (95%CI 1.02–1.60) for small for gestational age.

**Conclusions:**

Hypertension modifies the association between migration and unfavourable pregnancy outcomes. Although migrant women had a lower risk of preterm birth and small for gestational age than Belgian women, in the presence of hypertension, their risk was significantly higher than Belgian women with the same conditions. Further research is needed to analyse the complex relationships between migration, social status, women’s living conditions, and perinatal outcome.

## Introduction

With its history and location at the heart of Europe, Belgium is a land of many migrations. Just in the recent decade, a quarter of all births have been to women of non-Belgian nationalities [1]. Inequalities in pregnancy outcomes among migrant women are well documented in the literature [2]. Migrant and native women present different perinatal outcomes, with the former often having an increased risk of poor outcomes, regularly associated with their unfavourable socio-economic status (SES) [3]. However, epidemiological studies also report conflicting findings regarding the association between women’s migration and pregnancy outcomes. Different outcomes present various risks in different source populations and in different receiving countries [2].

In Belgium, the complex relationship between migration, socio-economic status (SES), and pregnancy outcomes has also been documented in various studies [4–6]. Compared with Belgian women, migrant women have an increased risk of perinatal mortality, which was mainly explained by the SES. For preterm birth (PTB) and low birth weight, they are higher in pregnancies of women from sub-Saharan Africa, but lower in women from North Africa compared to Belgian women [7].

Medical conditions including obesity, pre-existing or pregnancy-induced hypertension, pre-existing or gestational diabetes, are associated with increased perinatal risks. We know that these medical conditions are more prevalent in certain populations, such as hypertension (HT) in sub-Saharan African women [8,9] or diabetes in women from North Africa [10]. However, few studies analysed the possible role of maternal comorbidities on the association between migration and perinatal outcomes [11,12].

Our objective was to explore whether maternal comorbidities modify migrant women’s risks, knowing that they can differ depending on the nationality group and lead to various associations with pregnancy outcomes. In order to improve the understanding of perinatal inequalities, we analysed the effect of comorbidities, such as hypertension (HT), obesity, and diabetes, on preterm birth (PTB) and small for gestational age (SGA) among migrant women.

## Methods

### Study population and data

This is an observational population-based study. We used data from birth certificates taken from the Belgian civil registration system which includes all babies born in Belgium between the 1^st^ of January 2010 and the 31^st^ December 2019. Medical data is registered at the hospital by midwives and gynaecologists, and socio-economic data is recorded by the civil registration service within a fortnight of the birth, as reported by the parent(s). In Belgium, two perinatal epidemiological centres, SPE for the Dutch-speaking region and CEpiP for the French-speaking region, are in charge of data quality and completeness, as catalogued in birth and death certificates [13]. We included all singleton live births from 24 to 42 weeks’ gestational age. In order to analyse SGA, we excluded multiple births and those under 24 weeks or more than 42 weeks, in line with international growth curves [14]. The final size of the study population was 1 200 417 as presented in the study flowchart, S1 Fig.

### Definitions of the exposures and outcomes

The migration status was defined as being of foreign nationality at the time of child birth. We bundled nationalities into the six most represented groups: Belgium, European Union (EU 27), Eastern Europe, North Africa, Sub-Saharan Africa, and the Middle East, S2 Table. Due to heterogeneity and small numbers, the nationality categories of “others” or “unknown” were not analysed.

The socio-demographic variables include the following: maternal age, parity, maternal level of education, if living with a partner or not, and number of incomes in the household.

Maternal age was categorised into three groups; under 20 years, 20–35 years old, over 35 years. Parity was categorised into three groups; nulliparity, 1 or 2 births, and 3 or more births (grand multiparous). The maternal level of education was collated into four categories: superior (university or higher education), secondary (completed secondary school), primary (completed primary school or less), and other or unknown. Single women were defined as living alone. Data on paternal and maternal occupational status were combined to derive the number of incomes in the household (two, one, or none). In case of a single women, the number of incomes could only vary between zero and one. Due to a significant proportion of missing or unknown values, a category “unknown” was generated.

Maternal comorbidities were defined by the presence (yes or no) of hypertension (HT), obesity and diabetes. HT and diabetes were reported during antenatal care by clinicians in hospital medical records. HT includes both preexisting and gestational HT, although most cases are likely pregnancy induced due to the low prevalence of HT in women of reproductive age. Diabetes included preexisting and gestational diabetes. Maternal Body Mass Index (BMI) was calculated using the weight and height of the women registered at the first antenatal consultation. We categorised the BMI as obese (> 30 kg/m²) and non-obese (≤ 30 kg/m²).

Perinatal outcomes were preterm birth (PTB) and small for gestational age (SGA). PTB was defined as gestational age under 37 completed weeks at birth and SGA as a birth weight under 10^th^ centile according to Intergrowth-21^st^ standards (IG) [14].

### Statistical analysis

We present the characteristics of the population as frequencies by nationality group. We used logistic regression to estimate the crude odds ratios (OR) and their Confidence Interval (95%CI) for associations between maternal nationality and PTB and SGA. Multivariable models were developed to assess the independent effects of SES, and of the available maternal comorbidities (HT, obesity and diabetes) as potential confounders or effect modifiers. In a first model, we adjusted for SES (maternal age, parity, education, single status and income), and in a second model, we adjusted for SES and the comorbidities. Interaction between comorbidities and maternal nationality were tested (Chi² for interaction). We report the adjusted estimates when the variable was a confounder and a stratified analysis when the variable was an effect modifier.

We present the odds ratios and 95%CI derived from the logistic regression models and the p-values for the Wald *χ*^2^ test. The significance level was set at α = 0.05, and all analyses were performed using Stata SE18 software.

### Ethics statement

This study and the use of related data were approved by the Institutional Review Board (IRB) of the Belgian Statistical Office (Statbel): reference number 2022/052. (https://stabel.fgov.be/sites/default/files/files/documents/Over%20Statbel/Microdata_EN.pdf). Participant consent is the responsibility of Statbel in accordance with Belgian legislation. (https://statbel.fgov.be/en/about-statbel/privacy/privacy-gdpr).

## Results

### Characteristics of the population by maternal nationality group in Belgium

Migrant women accounted for 23.1% of all births, with 10.1% from EU27, 1.7% from East Europe, 4.0% from North Africa, 3.1% from Sub-Saharan Africa, and 2.4% from the Middle East. Migrant women were more socio-economically disadvantaged than Belgian women (Table 1). They were more likely to have completed only primary school (35–42% for East European, North African, Sub-Saharan African, and Middle Eastern women, versus 11% for Belgian women). Sub-Saharan African and East European women had the highest proportion of zero income in the household (40.2% and 34.4%, respectively). Sub-Saharan African women were more likely to be single women (40.6%). Conversely, Belgian women had the highest proportion of higher education (44.7%) and two incomes in the household (65.5%). A higher percentage of Sub-Saharan African women had HT (7.2%) and obesity (18.1%) compared to the other groups. North African women had the highest percentage of diabetes (12.6%).

**Table 1:** Characteristics of the population by maternal nationality group in Belgium, 2010-2019.

	All Births	Belgium	EU27	East Europe	North Africa	Sub-Saharan Africa	Middle East
*n* (%)	1 200 417	916 737 (76.9)	120 082 (10.1)	20 090 (1.7)	48 018 (4.0)	37 197 (3.1)	28 061 (2.4)
***Socio-demographic data***							
**Maternal age (year)**							
Under 20	31 540 (2.6)	21953 (2.4)	3901 (3.3)	1 838 (9.1)	679 (1.4)	1 266(3.4)	1 138 (4.1)
20–35	1 003 717 (83.6)	778 912 (85.0)	92 409 (77.0)	16 149 (80.4)	38 458 (80.1)	30 323 (81.5)	23 614 (84.2)
Over 35	165 160 (13.8)	115 872 (12.6)	23 772 (19.8)	2103 (10.5)	8881 (18.5)	5608 (15.1)	3309 (11.8)
**Single women**	155 772 (13.1)	110 183 (12.1)	16 604 (14.0)	3 830 (19.5)	3 460 (7.3)	14 793 (40.6)	3 011 (10.9)
**Parity**							
0	522 479 (43.6)	408 264 (44.6)	54 003 (45.0)	7 646 (38.1)	17 332 (36.1)	12 178 (32.8)	9 437 (33.6)
1-2	583 734 (48.7)	444 200 (48.5)	57 051 (47.6)	9 417 (46.9)	24 814 (51.7)	19 151 (51.5)	14 894 (53.1)
≥ 3	93 070 (7.8)	63 414 (6.9)	8 876 (7.4)	3 009 (15.0)	5 838 (12.2)	5 839 (15.7)	3 710 (13.2)
**Maternal education**							
Primary	178 953 (14.9)	100 946 (11.0)	20 827 (17.3)	7058 (35.1)	20 361 (42.4)	13 557 (36.5)	10 419 (37.1)
Secondary	397 657 (33.1)	317 886 (34.7)	28 234 (31.8)	4817 (24.0)	12 755 (26.6)	9 959 (26.8)	6 925 (24.7)
Superior	479 722 (40.0)	409 991 (44.7)	44 394 (37.0)	3 170 (15.8)	5 560 (11.6)	5 208 (14.0)	3 459 (12.3)
Unknown	144 085 (12.0)	87 914 (9.6)	16 627 (13.9)	5 045 (25.1)	9 342 (19.5)	8 473 (22.8)	7 258 (25.9)
**Number of incomes in the household**							
None	153 724 (12.8)	93 634 (10.2)	13 722 (11.4)	6 914 (34.4)	11 304 (23.5)	14 943 (40.2)	8 395 (29.9)
One	277 320 (23.1)	171 982 (18.8)	35 693 (29.7)	7 104 (35.4)	26 705 (55.6)	12 742 (34.3)	12 439 (44.3)
Two	681 429 (56.8)	600 635 (65.5)	59 734 (49.7)	2 611 (13.0)	4 530 (9.4)	4 115 (11.1)	2 690 (9.6)
Unknown	87 944 (7.3)	50 486 (5.5)	10 933 (9.1)	3461 (17.2)	5479 (11.4)	5397 (14.5)	4537 (16.2)
***Comorbidities***							
**Hypertension**	54 752 (4.6)	44 441 (4.9)	4 271 (3.6)	543 (2.7)	1 092 (2.3)	2 673 (7.2)	675 (2.4)
**Diabetes**	71 740 (6.0)	49 526 (5.4)	7 353 (6.2)	1 022 (5.2)	6 003 (12.6)	3 323 (9.0)	2 190 (7.9)
**Obesity**	142 957 (12.7)	111 980 (12.9)	12 015 (10.8)	1 575 (8.8)	6 124 (14.3)	5 937 (18.1)	2 995 (12.0)
***Perinatal outcome***							
**Preterm birth**	76269 (6.4)	60 386 (6.6)	7 007 (5.8)	1 054 (5.2)	2 015 (4.2)	2 482 (6.7)	1 499 (5.3)
**SGA**	77 411 (6.5)	59 386 (6.5)	7557 (6.3)	1171 (5.8)	2495 (5.2)	2825 (7.6)	1968 (7.0)

The risk of PTB in Belgian women between 2010 and 2019 was 6.6%. Overall, non-Belgian women had a lower risk of PTB than Belgians, except for Sub-Saharan African women (6.7%). Sub-Saharan women had a higher percentage of SGA babies (7.6%) compared to the other groups.

### Risk factors for PTB and SGA in Belgium

Socio-demographic variables were significantly associated with PTB and SGA (p < 0.001) (Table 2). Belonging to a low socio-economic level, being younger (<20 years old) and older (>35 years old), single, less educated, and without any income in the household, significantly increased the odds ratio of PTB and SGA. The OR of PTB and SGA was significantly higher among primiparas and in grand multiparas.

**Table 2:** Risk factors for Preterm Birth and SGA in Belgium, frequencies and Odds Ratios (ORs) (95% CI).

	Preterm Birth			SGA		
	**n (%)**	**OR (CI 95%)**	**p-value**	**n (%)**	**OR (CI 95%)**	**p-value**
**Maternal age (years)**			<0.001			<0.001
Under 20	2564 (8.1)	1.35 (1.29–1.41)		3401 (10.8)	1.80 (1.74–1.87)	
20–35	61 761 (6.2)	1 (Ref.)		63 196 (6.3)	1 (Ref.)	
Over 35	12 070 (7.3)	1.20 (1.18–1.23)		10 814 (6.5)	1.04 (1.02–1.06)	
**Single mother**	13 358 (8.6)	1.47 (1.44–1.49)	<0.001	15 225 (9.8)	1.72 (1.69–1.75)	<0.001
**Parity**			<0.001			<0.001
0	38 043 (7.3)	1.41 (1.39–1.43)		43 231 (8.3)	1.76 (1.73–1.78)	
1–2	30 856 (5.3)	1 (Ref.)		28 437 (4.9)	1 (Ref.)	
≥ 3	7429 (8.0)	1.55 (1.51–1.60)		5681 (6.1)	1.27 (1.23–1.30)	
**Maternal education**			<0.001			<0.001
Primary	12 440 (7.0)	1.28 (1.26–1.31)		14 784 (8.3)	1.79 (1.75–1.83)	
Secondary	26 875 (6.8)	1.25 (1.23–1.27)		28 671 (7.2)	1.54 (1.51–1.57)	
Superior	26 327 (5.5)	1 (Ref.)		23 017 (4.8)	1 (Ref.)	
Unknown	10 753 (7.5)	1.39 (1.35–1.42)		10 939 (7.6)	1.63 (1.59–1.67)	
**Number of incomes in the household**			<0.001			<0.001
None	11 922 (7.8)	1.33 (1.31–1.36)		14 579 (9.5)	1.86 (1.82–1.89)	
One	17 740 (6.4)	1.08 (1.06–1.10)		19 715 (7.1)	1.36 (1.33–1.38)	
Two	40 318 (5.9)	1 (Ref.)		36 428 (5.3)	1 (Ref.)	
Unknown	6415 (7.3)	1.25 (1.21–1.28)		6689 (7.6)	1.46 (1.42–1.50)	
**Hypertension**	9456 (17.3)	3.38 (3.30–3.46)	<0.001	7228 (13.2)	2.34 (2.28–2.40)	<0.001
**Diabetes**	6612 (9.2)	1.55 (1.51–1.59)	<0.001	4185 (5.8)	0.90 (0.87–0.92)	<0.001
**Obesity**	8867 (6.2)	1.03 (1.01–1.06)	<0.05	7159 (5.0)	0.75 (0.74–0.77)	<0.001
**Nationality**			<0.001			<0.001
**Belgium**	60 386 (6.6)	1 (Ref.)		59 386 (6.5)	1 (Ref.)	
**Migrant**	14 057 (5.6)	0.83 (0.82–0.85)		16 016 (6.3)	0.97 (0.96–0.99)	
EU27	7007 (5.8)	0.88 (0.86–0.90)		7557 (6.3)	0.97 (0.95–0.99)	
East Europe	1054 (5.3)	0.79 (0.74–0.84)		1171 (5.8)	0.89 (0.84–0.95)	
North Africa	2015 (4.2)	0.62 (0.59–0.65)		2495 (5.2)	0.79 (0.76–0.82)	
Sub–saharan Africa	2482 (6.7)	1.01 (0.97–1.06)		2825 (7.6)	1.19 (1.14–1.23)	
Middle East	1499 (5.3)	0.80 (0.76–0.84)		1968 (7.0)	1.10 (1.04–1.14)	

Hypertension was strongly associated with PTB (OR 3.38; 95%CI 3.30–3.46) and SGA (OR 2.34; 95%CI 2.28–2.40). Obesity and diabetes were associated with perinatal outcomes, with a significantly increased OR for PTB but with a lower OR of SGA (p < 0.001).

Migrant women had a significantly lower OR of PTB compared to Belgian women (p < 0.001), except women from sub-Saharan Africa, who presented a similar risk. A similar pattern was observed for SGA, with a lower OR for migrant women, except women from sub-Saharan Africa and the Middle East, for whom the OR was higher compared to Belgian women.

### Association between maternal nationality and, PTB and SGA, stratified by hypertension

Given the significant modifying effect of HT (p < 0.001) on the association between nationality and perinatal outcomes, we stratified our models according to the presence or absence of HT (Table 3, and Table 4). The PTB OR for women without HT was significantly lower among all migrant women than among Belgian women (p < 0.001), even after adjustment for SES and co-morbidities (Table 3). In contrast, for women with HT, migrant women presented a higher OR of PTB. After adjustment for SES and comorbidities, the OR of PTB was significantly higher for women from Sub-Saharan Africa, and the Middle East compared to Belgian women: 1.45 (1.30–1.62) and 1.24 (1.01–1.54), respectively.

**Table 3:** Association between maternal nationality and Preterm Birth stratified by hypertension: OR and adjusted aOR (95%CI).

	OR (CI 95%)	aOR^1^ (IC 95%)	aOR^2^ (CI 95%)	OR (CI 95%)	aOR^1^ (CI 95%)	aOR^2^ (CI 95%)
	**without hypertension**			**with hypertension**		
**Belgium**	1 (Ref.)			1 (Ref.)		
**EU27**	0.88	0.84	0.82	1.07	1.03	1.00
	(0.85-0.90)	(0.81-0.86)	(0.80-0.85)	(0.99-1.17)	(0.94-1.12)	(0.91-1.09)
**East Europe**	0.78	0.67	0.70	1.19	1.07	0.96
	(0.73-0.83)	(0.63-0.72)	(0.65-0.75)	(0.96-1.48)	(0.86-1.33)	(0.75-1.22)
**North Africa**	0.61	0.55	0.53	1.33	1.20	1.10
	(0.58-0.64)	(0.53-0.58)	(0.50-0.56)	(1.15-1.54)	(1.02-1.40)	(0.93-1.31)
**Sub-Saharan Africa**	0.83	0.69	0.67	1.83	1.56	1.45
	(0.79-0.87)	(0.65-0.72)	(0.63-0.70)	(1.67-2.00)	(1.42-1.72)	(1.30-1.62)
**Middle East**	0.80	0.73	0.71	1.36	1.26	1.24
	(0.76-0.85)	(0.68-0.77)	(0.67-0.75)	(1.13-1.64)	(1.04-1.53)	(1.01-1.54)

^1^Adjusted for SES: maternal age, parity, number of incomes, maternal education, single mother

^2^Adjusted for SES and co-morbidities: obesity, diabetes

**Table 4:** Association between maternal nationality and SGA stratified by hypertension: OR and adjusted aOR (95%CI).

	OR (CI 95%)	aOR^1^ (CI 95%)	aOR^2^ (CI 95%)	OR (CI 95%)	aOR^1^ (CI 95%)	aOR^2^ (CI 95%)
	**without Hypertension**			**with Hypertension**		
**Belgium**	1 (Ref.)			1 (Ref.)		
**EU27**	0.97	0.89	0.88	1.12	1.06	1.03
	(0.95-1.00)	(0.86-0.91)	(0.85-0.90)	(1.02-1.23)	(0.96-1.16)	(0.93-1.14)
**East Europe**	0.89	0.68	0.64	1.26	1.06	0.95
	(0.84-0.95)	(0.64-0.72)	(0.60-0.69)	(1.00-1.59)	(0.83-1.35)	(0.73-1.24)
**North Africa**	0.79	0.62	0.62	1.36	1.13	1.08
	(0.76-0.83)	(0.59-0.65)	(0.59-0.65)	(1.16-1.60)	(0.96-1.34)	(0.89-1.30)
**Sub-Saharan Africa**	1.13	0.83	0.84	1.35	1.19	1.17
	(1.08-1.18)	(0.80-0.87)	(0.80--0.88)	(1.21-1.50)	(1.06-1.34)	(1.03-1.32)
**Middle East**	1.10	0.88	0.86	1.42	1.29	1.28
	(1.05-1.16)	(0.83-0.92)	(0.81-0.90)	(1.16-1.73)	(1.04-1.58)	(1.02-1.60)

^1^Adjusted for SES: maternal age, parity, number of incomes, maternal education, single mother

^2^Adjusted for SES and co-morbidities: obesity, diabetes

For SGA, a similar pattern was observed (Table 4). All migrant women without HT had a significantly lower adjusted OR of SGA than Belgian women (p < 0.001). Sub-Saharan African and Middle Eastern women had higher OR of SGA compared to Belgian in univariable analysis: 1.13 (1.08–1.18) and 1.10 (1.05–1.16), respectively. However, after adjustment for SES and comorbidities, their OR was lower compared to Belgian women: 0.84 (0.80–0.88) and 0.86 (0.81–0.90), respectively. For women with HT, all migrant women presented a higher OR of SGA compared to Belgian women. After adjustment the excess risk for women from Sub-Saharan Africa and the Middle East remained significantly higher than for Belgian women.

## Discussion

This study shows that hypertension is an effect modifier on the association between migration and both PTB and SGA. Although hypertension increases the risk of PTB and SGA in all women, the risk was even higher among migrant women. There was a reduced risk of PTB and SGA in normotensive migrant women compared to normotensive Belgian women, and an increased risk of PTB and SGA for hypertensive migrant women, compared to Belgian women presenting the same conditions.

### Protective effect of migration among non-hypertensive women

Our results concur with several studies that have shown that migrant women have favourable pregnancy outcomes despite their unfavourable SES conditions [2,15]. This is known as the epidemiological paradox in migrant health (the “healthy migrant effect”) [16]. A number of hypotheses have been suggested to explain this incongruity. People in good health may be more likely to decide to migrate [17]. Migrant women may have retained healthier habits from their country of origin, such as eating less processed food, and avoiding drugs, tobacco and alcohol [11]. Another explanation could lie in the resilience linked to migratory experiences and the mental strength of migrant women, which may both translate into better health [18,19].

### Increased rate of PTB and SGA in hypertensive migrant women

Hypertension is a known factor of significant risks of obstetric and neonatal morbidity. This contributes to a higher complication risks, and has been well described for women of African or Asian origins [8]. An increased risk of adverse neonatal outcomes (PTB, SGA, lower Apgar score, assisted ventilation, seizures, or death) has been shown to be associated with ethnic origins among women with chronic HT [20]. It has been suggested that the different outcomes may be due to the different pathophysiological pathways leading to PTB and SGA in migrant women, because of underlying genetic differences in the severity of their HT [21]. This may in turn explain the interaction between HT and migration shown in our study. Sub-Saharan women are known to have a higher risk of severe preeclampsia [22].

Explanatory hypotheses include barriers to accessing healthcare, communication issues, knowledge of the healthcare provider, systemic racism, living conditions and much more.

### Health care among migrant

Migrant women have less good access and underuse the health care services, leading to inadequate or delayed care. They suffer financial hardship, are faced with complex administrative procedures and/or have no access to social security coverage at all [23]. In Belgium, women who are not eligible for the social security coverage still have the right to access perinatal care free of charge, by applying for ‘emergency healthcare coverage’. However, women with emergency coverage were found to enter antenatal care later than women with regular health insurance [24].

Language barriers and health literacy are a crucial obstacle to adequate care. In a study of a migrant population in the United States, about half of the participants reported that they did not fully understand the medical information they received [25]. According to recent studies, language barriers have an impact on access to care and on the adequacy of antenatal follow-up. They increase the risk of unfavourable perinatal outcomes [26,27].

The excess risk of PTB and SGA among migrant women with HT can be explained by a difference in care between migrant and native women. In France, a study showed that medical procedures are different, and that hypertensive women from Sub-Saharan Africa are less likely to use healthcare (7). This has been described as implicit biases due to systemic racism [28]. In the US, a qualitative study on antenatal care found that migrant women perceived their care as disrespectful and very stressful [29]. More recently, health inequality has been called to account in reports showing a higher perinatal risk for women of African or Asian origin in Western countries such as France, the US, and the UK. These reports highlight the discrimination, or even racism, suffered by these women [30–32].

### Lifestyle conditions

In addition, lifestyle can bring together confounding factors. HT is often associated to individual’s lifestyle factors. as social isolation, poor living and working conditions, stressful life events related to migration and discrimination. This should also be studied as they may explain part of our results [33]. Moreover, it has been shown that among US African-American women, metabolic diseases were perceived as being “black diseases”. This conceptualisation is maintained over generations [34–36]. It, therefore leads to the minimisation of the critical roles played by lifestyle, diet, and appropriate eating behaviours in this population, all of which are central to fighting diseases, such as HT, that have an impact on pregnancy outcomes. One hypothesis is that a similar phenomenon is being observed among migrant groups in Belgium.

The accumulation of all these factors may interact and be making hypertensive migrant women more vulnerable compared to the Belgian population.

### Strengths and limitations

The key strength is the novelty of the main finding: the effect of HT on pregnancy outcome differs according to the geographical origin of the pregnant woman. If this is confirmed in other studies it has direct implications.

Another strength of this study is that it used an exhaustive national dataset including undocumented, refugee and homeless women, who are often absent from administrative databases, and including demographic, socio-economic and clinical factors.

The use of population registers makes it possible to analyse a very large number of cases. However, these registers often lack precision in the clinical evaluation of each pathology. In our study, the limitations arise in particular from the coding of comorbidities available in the Belgian register. For HT, we lacked clinical information, such as whether HT preceded the pregnancy or not, or the severity of the HT and its complications such as pre-eclampsia. For diabetes, a distinction between pre-gestational and gestational diabetes would have been interesting.

As migration encompasses different statuses [37], the heterogeneity of the group of migrants studied limits the possible comparisons with data obtained in other studies. It would have been interesting to know the length of stay in the host country. This could have a negative impact on medical conditions through the acculturation process [17,38].

Furthermore, information on healthcare access and perinatal healthcare use might allow for a better understanding of the nature of health inequalities [39].

### Potential future directions for research

The modified effect of country of origin on the perinatal outcomes in women with HT generates new research hypotheses, in particular the complex relationships between migration, social status, life experience, maternal conditions and perinatal outcomes. These can be addressed by ad hoc quantitative study with information related to social circumstances and utilisation of services. They will also require qualitative studies involving migrant families and health care providers.

In the meantime, from an operational viewpoint, it is probably acceptable to consider that, in Belgium at least, hypertensive pregnant women from Sub-Saharan Africa and the Middle East constitute an increased risk population, and to monitor possible change.

## Conclusion

Although migrant women have a lower risk of PTB and SGA than Belgian women, in the presence of HT, their risk is significantly higher than that of Belgian women presenting the same comorbidities. This reinforces the findings according to which pregnancy outcomes differ per nationality groups, and provides insights into the role played by medical factors. This study enables us to identify groups of migrant women at risk of PTB and SGA, particularly hypertensive Sub-Saharan African and Middle Eastern women, in order to optimise their medical care.

## Supporting information

S1 Fig This is the Study flowchart. (DOCX)

S2 Table This is the List of countries included in each nationality category. (DOCX)
