# Supplementary material for: Hypertension as an effect modifier for preterm and small for gestational age births in migrant women in Belgium: A population-based study
Source: PLoS One. 2025 May 14;20(5):e0323652. doi: 10.1371/journal.pone.0323652 (PMC12077694; doi:10.1371/journal.pone.0323652)
Supplement: S1 Fig — (DOCX) [file pone.0323652.s001.docx]

**S1 Fig. Study flowchart.**
