# Supplementary material for: Hypertension as an effect modifier for preterm and small for gestational age births in migrant women in Belgium: A population-based study
Source: PLoS One. 2025 May 14;20(5):e0323652. doi: 10.1371/journal.pone.0323652 (PMC12077694; doi:10.1371/journal.pone.0323652)
Supplement: S2 Table — (DOCX) [file pone.0323652.s002.docx]

**S2 Table.** List of countries included in each nationality category, *UK included as Brexit was effective in 2021

| ***EU27*** | | ***East Europe and Russia*** | ***North Africa*** | ***Sub-Saharan Africa*** | | ***Middle East*** | |
| --- | --- | --- | --- | --- | --- | --- | --- |
| Austria |  | Albania | Algeria | Angola | Liberia | Afghanistan | United Arab Emirates |
| Bulgaria | Slovenia | Belarus | Egypt | Benin | Madagascar | Armenia | Uzbekistan |
| Croatie | Spain | Bosnia-Herzegovina | Libya | Botswana | Malawi | Azerbadjan | Yemen |
| Cyprus | Sweden | Kosovo | Mauritania | Burkina Faso | Mali | Bahrain |  |
| Czech Republic | United Kingdom* | Macedonia | Morocco | Burundi | Mauritius | Georgia |  |
| Denmark |  | Moldova | Tunisia | Cabo Verde | Mozambique | Iran |  |
| Estonia |  | Montenegro |  | Cameroon | Namibia | Iraq |  |
| Finland |  | Republic of Moldova |  | Central African Republic | Ngwane | Israel |  |
| France |  | Russian Federation |  | Chad | Niger | Jordan |  |
| Germany |  | Serbia |  | Comoros | Nigeria | Kazakhstan |  |
| Greece |  | Ukraine |  | Democratic Rep. of Congo | Rwanda | Kuwait |  |
| Hungary |  |  |  | Djibouti | Sao Tome | Kyrgistan |  |
| Ireland |  |  |  | Equatorial Guinea | Senegal | Lebanon |  |
| Italy |  |  |  | Eritrea | Seychelles | Oman |  |
| Latvia |  |  |  | Ethiopia | Sierra Leone | Pakistan |  |
| Lithuania |  |  |  | Gabon | Somalia | Palestine |  |
| Luxembourg |  |  |  | Gambia | South Africa | Saudi Arabia |  |
| Malta |  |  |  | Ghana | Sudan | Syria |  |
| Netherlands |  |  |  | Guinea | Tanzania | Tajikistan |  |
| Poland |  |  |  | Guinea-Bissau | Togo | Turkey |  |
| Portugal |  |  |  | Ivory coast | Uganda | Turkmenistan |  |
| Romania |  |  |  | Kenya | Zambia |  |  |
| Slovakia |  |  |  | Lesotho | Zimbabwe |  |  |
